# The use of benzodiazepines and Z-drugs in the Acute Psychiatric Unit at Cavan General Hospital

**DOI:** 10.1192/bjo.2021.818

**Published:** 2021-06-18

**Authors:** Salah Ateem, Rachael Cullivan

**Affiliations:** 1 St. Davnet's Hospital; 2 Cavan General Hospital

## Abstract

**Aims:**

Benzodiazepines and Z-drugs are used frequently in acute psychiatric wards, however long-term administration can result in undesirable consequences. Guidelines recommend prescription of the lowest effective dose for the shortest period and if possible to prescribe “as required” rather than regularly. The 25-beded inpatient unit at Cavan General Hospital admits adult patients requiring acute care from the counties of Cavan and Monaghan. Admissions are accepted from four community mental health teams, two psychiatry of old age teams and the rehabilitation and mental health of intellectual disability teams. In order to evaluate the potential to improve our practice of prescribing benzodiazepine and Z-drugs, it was decided to evaluate current use.

**Method:**

The NICE guidelines were consulted, and we retrospectively reviewed the use of these agents from mid-January to the end of May 2020. Demographic variables included age, gender, and county. Patients were stratified into three groups, the benzodiazepine group, the Z-drugs group, and the combined benzodiazepine and Z-drugs group. In each group therapeutic variables were recorded including the medication type, dose, frequency, prescriber, and duration of treatment. Other variables included psychiatric diagnoses, length of inpatient admission, status on admission, and recommendations on discharge

**Result:**

There were 101admissions during that period, and 74 of them were prescribed these agents (n = 74; 73.3%). Fifty one (n = 51; 68.9%) received benzodiazepines only, twenty-three (n = 23; 31.1%) were prescribed Z-drugs, and twelve (n = 12; 16.2%) received both benzodiazepines and Z-drugs. Forty two patients (n = 42; 56.8%) were commenced on hypnotics in the APU, 23 patients (n = 23; 31.1%) already received hypnotics from the CMHTs, and the rest were prescribed by both. Thirty two patients (n = 32; 43.2%) were discharged on hypnotics. Patients admitted involuntarily and female patients had longer admissions (mean of 16.62 ± 3.26 days and 16.16 ± 2.89 days respectively). Schizophrenia and BPAD were the commonest diagnoses.

**Conclusion:**

It appears that large amounts of these agents are used in the Acute Hospital Setting which is not overly surprising given the severity of illness and clinical indications however improved awareness could still lead to more appropriate and hopefully reduced use. We therefore recommend:

A formal audit including appropriate interventions i.e., educate staff and patients, highlight guidelines, and review subsequent practice.

Train staff in safer prescribing practices including prn rather than regular use if appropriate.

Regularly review discharge prescriptions indicating recommended duration of use.

